# Incidence and Diversity of Torix *Rickettsia*–Odonata Symbioses

**DOI:** 10.1007/s00248-020-01568-9

**Published:** 2020-08-07

**Authors:** Panupong Thongprem, Helen R. Davison, David J. Thompson, M. Olalla Lorenzo-Carballa, Gregory D. D. Hurst

**Affiliations:** 1 Institute of Infection, Veterinary and Ecological Sciences, University of Liverpool, Liverpool, L69 7ZB UK; 2 ECOEVO Group, Universidade de Vigo, E.E. Forestal, Campus Universitario A Xunqueira, 36005 Pontevedra, Spain

**Keywords:** Torix, *Rickettsia*, Odonates, Endosymbionts

## Abstract

Heritable microbes are an important component of invertebrate biology, acting both as beneficial symbionts and reproductive parasites. Whilst most previous research has focussed on the ‘*Wolbachia* pandemic’, recent work has emphasised the importance of other microbial symbionts. In this study, we present a survey of odonates (dragonflies and damselflies) for torix group *Rickettsia*, following previous research indicating that this clade can be common in other aquatic insect groups. PCR assays were used to screen a broad range of odonates from two continents and revealed 8 of 76 species tested were infected with *Rickettsia*. We then conducted further deeper screening of UK representatives of the Coenagrionidae damselfly family, revealing 6 of 8 UK coenagrionid species to be positive for torix *Rickettsia*. Analysis of *Rickettsia* gene sequences supported multiple establishments of symbiosis in the group. Some strains were shared between UK coenagrionid species that shared mtDNA barcodes, indicating a likely route for mitochondrial introgression between sister species. There was also evidence of coinfecting *Rickettsia* strains in two species. FISH analysis indicated *Rickettsia* were observed in the ovarioles, consistent with heritable symbiosis. We conclude that torix *Rickettsia* represent an important associate of odonates, being found in a broad range of species from both Europe and South America. There is evidence that coinfection can occur, vertical transmission is likely, and that symbiont movement following hybridisation may underpin the lack of ‘barcoding gap’ between well-established species pairs in the genus. Future work should establish the biological significance of the symbioses observed.

**Electronic supplementary material:**

The online version of this article (10.1007/s00248-020-01568-9) contains supplementary material, which is available to authorized users.

## Introduction

Animals and plants commonly form associations with microbes, either by interacting with environmental microbes on their surface, in their gut, or with microbes living inside the organism’s tissues as endosymbionts. A subset of these may pass vertically from a female to her offspring and are termed heritable symbionts. Vertical transmission aligns the fitness interest of host and symbiont and has selected for these microbes to play important roles in host function. Carrying a symbiont can influence a host individual’s reproductive success [1–3], modulate its ability to defend against natural enemies [4], or alter digestion and nutrient production [5, 6]. However, the maternal inheritance of symbionts creates a dependence of symbiont fitness solely on the production and survival of daughters, leading to the evolution of reproductive parasitic phenotypes [2, 7].

The best-known example of a heritable symbiont is *Wolbachia*, which is estimated to infect over 50% of insect species [8]. *Wolbachia* is most commonly known as a reproductive manipulator, which drives itself into a population through cytoplasmic incompatibility, feminisation, male killing and induced parthenogenesis [3, 7, 9]. In some cases, like the butterfly *Acraea encedon*, it can result in highly female-biassed population sex ratios that alter mating behaviour [10]. *Wolbachia* can also act as a nutritional symbiont for blood-feeding insects by synthesising B vitamins that the host cannot make on its own or obtain from its diet, and can enhance tolerance to RNA virus infection in diverse species [11]. This covers just a few examples of *Wolbachia* impacts and is not an exhaustive list.

Whilst *Wolbachia* is not the only bacterial symbiont of insects, it is the best studied associate of terrestrial and, to a lesser extent, freshwater taxa [12]. The documentation for endosymbionts of freshwater insects is particularly poor when compared with terrestrial insects, with the notable exception of mosquitoes [13]. Recently, the presence of torix group *Rickettsia* (hereafter referred to as torix *Rickettsia*) has been noted in a variety of aquatic invertebrate taxa. First discovered in *Torix* leeches [14, 15], hotspots of torix *Rickettsia* have been observed in *Culicoides* biting midges [16], deronectid diving beetles [17] and dolichopodid flies [18]. To date, the impact of symbionts from this group on host biology is unclear, with the exception of bark lice, in which *Rickettsia* infection is associated with parthenogenetic reproduction by the host [19]. Analysis of the symbiont genome sequences from midges found no evidence for B vitamin synthesis capacity [16]. However, the symbiont infection is a potentially important aspect of biology that has generally been overlooked in many aquatic insects.

The cosmopolitan insect order Odonata (dragonflies and damselflies, generally referred to as ‘odonates’) are associated with freshwater habitats. This ecologically important taxon of insects is easily identifiable, enabling their use in citizen science and in conservation as indicator species for monitoring the health of freshwater habitats [20]. They have also been identified as model organisms in ecological and evolutionary research [21]. Odonates are predatory insects with aquatic larvae and aerial adults, which depend on freshwater habitats in all stages of life. These insects have recently been revealed as hosts for *Wolbachia* [22–24], but surveys for other members of the *Rickettsiales* have yet to be completed. Investigating other symbiotic interactions in these ecologically important species could help enrich biological and ecological knowledge of both symbiotic bacteria and odonate hosts. Exploratory research will hopefully encourage further studies in this aspect of insect-endosymbiont evolution.

In this study, we first present an analysis of the incidence of *Rickettsia* infection in odonates through PCR assays. The screened species combined a broad sweep of biogeographical and taxonomic diversity. We also explored infection in-depth with a greater number of individuals in the damselfly family Coenagrionidae in the UK, which were readily available for collection. We performed FISH analysis of *Rickettsia* tropism in *Coenagrion puella* to establish if the symbiont is present in developing oocytes and thus determine the likelihood of vertical transmission.

## Methods

### Sample Collection and DNA Preparation

Existing odonate DNA from previous studies [25–32] and freshly collected leg material were tested for the presence of *Rickettsia*. Where leg material was obtained, a Promega Wizard® Genomic DNA Purification kit was used for DNA preparation. The analysed material covered a total of 284 individuals from 76 species within 8 families, from the UK, South America, mainland Europe and the Azores (Table 1). To enable a view of the commonness within species and any sex bias in presence, a focussed screening of 112 individuals belonging to 8 damselfly species within the family Coenagrionidae from the UK was executed in further depth, which included 5 additional species than the broad screen (Table 1).

**Table 1:** Screening results split according to screen type. The broad screen includes species from across South America, continental Europe, the Azores and the UK. The UK species included in the broad screen were tested prior to the focused screen and were used as the basis for doing the focussed screen. The focused screen covers coenagrionid species from the UK in greater breadth and depth

No.	Species	Family	Location	Number infected (number tested)
Broad global screen results				
Suborder Anisoptera (dragonflies)				
1	*Anax imperator*	Aeshnidae	Italy, Spain, Azores and continental Portugal	0 (14)
2	*Oxygastra curtisii*	Corduliidae	Tojal, Portugal	0 (1)
3	*Cannaphila vibex*	Libellulidae	Maquipucuna, Ecuador	0 (1)
4	*Erythrodiplax amazonica*	Libellulidae	Tiputini Ecuador	0 (1)
5	*E. kimminsi*	Libellulidae	Tiputini Ecuador	0 (3)
6	*E. unimaculata*	Libellulidae	Tiputini Ecuador	0 (1)
7	***Libellula depressa***	Libellulidae	Cheshire, UK	1 (1)
8	*Orthemis cultriformis*	Libellulidae	Tiputini, Ecuador	0 (1)
9	***Sympetrum fonscolombii***	Libellulidae	Azores, Portugal; Sardinia, Italy	2 (22)
10	*Trithemis annulata*	Libellulidae	Pontevedra, Spain	0 (1)
Suborder Zygoptera (damselflies)				
11	*Calopteryx haemorrhoidalis*	Calopterygidae	Italy, Portugal, Spain	0 (8)
12	*C. splendens*	Calopterygidae	Frosinone, Italy	0 (2)
13	*Haetarina* sp*.*	Calopterygidae	Peru	0 (1)
14	*Aeolagrion* sp.	Coenagrionidae	Pará, Brazil	0 (1)
15	*A. axine*	Coenagrionidae	Napo, Ecuador	0 (3)
16	*A. quadratum*	Coenagrionidae	Xalapa Mexico	0 (3)
17	*A. inca*	Coenagrionidae	Pacaya-Samiria, Loreto, Peru	0 (1)
18	*Argia joergenseni*	Coenagrionidae	Argentina	0 (2)
19	*A. kokama*	Coenagrionidae	Tiputini, Ecuador	0 (1)
20	*Bromeliagrion* sp.	Coenagrionidae	Pará, Brazil	0 (1)
21	*B. fernandezianum*	Coenagrionidae	Tiputini Ecuador	0 (1)
22	*B. renhi*	Coenagrionidae	Tiputini, Ecuador	0 (1)
23	***Coenagrion puella***	Coenagrionidae	Cheshire, UK	28 (28)
24	***Enallagma cyathigerum***	Coenagrionidae	Cheshire, UK	7 (7)
25	*Ischnura elegans*	Coenagrionidae	Cheshire, UK	0 (10)
26	*I. graellsii*	Coenagrionidae	Galicia	0 (18)
27	*I. hastata*	Coenagrionidae	Azores (Portugal), Dominican Republic, Jamaica, Cuba, Mexico, Florida	0 (43)
28	*Leptobasis vacillans*	Coenagrionidae	Santiago de Cuba, Cuba	0 (2)
29	*Metaleptobasis brysonima*	Coenagrionidae	Pará, Brazil	0 (1)
30	*M. mauffrayi*	Coenagrionidae	Tiputini, Ecuador	0 (3)
31	*M. quadricornis*	Coenagrionidae	Pará, Brazil	0 (1)
32	*Phoenicagrion karaja*	Coenagrionidae	Pará, Brazil	0 (3)
33	***Pyrrhosoma nymphula***	Coenagrionidae	Cheshire, UK	1 (2)
34	*Telebasis carmesina*	Coenagrionidae	Minas Gerais, Brazil	0 (1)
35	*T. dominicana*	Coenagrionidae	Represa Chalons, Cuba	0 (3)
36	*T. salva*	Coenagrionidae	Morelos, México	0 (2)
37	*Heteragrion bariai*	Megapodagrionidae	Napo, Ecuador	0 (1)
38	*Hypolestes clara*	Megapodagrionidae	Jamaica	0 (12)
39	*H. hatuey*	Megapodagrionidae	Arroyo Bermejo, Dominican Republic	0 (10)
40	*H. trinitatis*	Megapodagrionidae	Cuba	0 (10)
41	*Oxystigma* sp.	Megapodagrionidae	Pará, Brazil	0 (1)
42	*Philogenia* sp.	Megapodagrionidae	Napo, Ecuador	0 (1)
43	*Chalcopteryx rutilans*	Polythoridae	Trocha Quebrada, Peru	0 (1)
44	*Cora* sp.	Polythoridae	Panguana, Peru	0 (1)
45	*Polythore aurora*	Polythoridae	Iquitos, Peru	0 (1)
46	***P. lamerceda***	Polythoridae	Peru	1 (3)
47	*P. ornata*	Polythoridae	Pampa Hermosa, Peru	0 (6)
48	***P. picta***	Polythoridae	Pozuzo, Peru	1 (7)
49	*P. spaeteri*	Polythoridae	Panguana, Peru	0 (4)
50	*P. victoria*	Polythoridae	Pozuzo, Peru	0 (9)
51	*Drepanoneura* sp.	Protoneuridae	Napo, Ecuador	0 (3)
52	***D. muzoni***	Protoneuridae	Tiputini, Ecuador	1 (2)
53	*Epipleoneura metallica*	Protoneuridae	Mato Grosso, Brazil	0 (3)
54	*E. fuscaenea*	Protoneuridae	Guyana	0 (2)
55	*E. humeralis*	Protoneuridae	Tiputini, Ecuador	0 (4)
56	*E. machadoi*	Protoneuridae	Mato Grosso, Brazil	0 (2)
57	*E. williamsoni*	Protoneuridae	Minas Gerais, Brazil	0 (1)
58	*Neoneura* sp.	Protoneuridae	Pará, Brazil	0 (2)
59	*N. amelia*	Protoneuridae	Veracruz Mexico	0 (1)
60	*N. bilinearis*	Protoneuridae	Guyana	0 (1)
61	*N. confudens*	Protoneuridae	Guyana	0 (2)
62	*N. denticulata*	Protoneuridae	Pará, Brazil	0 (1)
63	*N. joana*	Protoneuridae	Guyana	0 (2)
64	*N. myrthea*	Protoneuridae	Guyana	0 (2)
65	*N. maria*	Protoneuridae	Cuba	0 (3)
66	***N. sylvatica***	Protoneuridae	Mato Grosso, Brazil	1 (2)
67	*Phasmoneura* sp.	Protoneuridae	Mato Grosso, Brazil	0 (1)
68	*P. exigua*	Protoneuridae	Mato Grosso, Brazil	0 (1)
69	*Protoneura* sp.	Protoneuridae	Pará, Brazil	0 (1)
70	*P. caligata*	Protoneuridae	Topes de Collantes, Cuba	0 (1)
71	*P. capillaris*	Protoneuridae	Dos Bocas, Cuba	0 (1)
72	*P. klugi*	Protoneuridae	Tiputini, Ecuador	0 (1)
73	*P. sanguinipes*	Protoneuridae	Dominican Republic	0 (3)
74	*P. viridis*	Protoneuridae	Jamaica	0 (1)
75	*Psaironeura* sp.	Protoneuridae	Pará, Brazil	0 (1)
76	*P. tenuissima*	Protoneuridae	Tiputini, Ecuador	0 (4)
Additional UK coenagrionid damselflies screened				
1	***Coenagrion mercuriale***	Coenagrionidae	Hampshire, UK	19 (30)
2	***C. pulchellum***	Coenagrionidae	Norfolk, UK	15 (20)
3	*Ceriagrion tenellum*	Coenagrionidae	Hampshire, UK	0 (5)
4	***Erythromma najas***	Coenagrionidae	Cheshire, UK	1(5)
5	***Pyrrhosoma nymphula***	Coenagrionidae	Cheshire, UK	4 (7)

Species positive for *Rickettsia* in the PCR assays are highlighted in bold

### General PCR Screening for *Rickettsia*

DNA was first quality checked (QC) to confirm that the samples contained amplifiable DNA template after storage/preparation. DNA QC was performed using the mtDNA barcoding primer pairs LCO_2190 (5′-GGT CAA CAA ATC ATC AAG ATA TTG G-3′)/HCO_2198 (5′-TAA ACT TCA GGG TGA CCA AAA AAT CA-3′) [33] and C1J_1718 (5′-GGA GGA TTT GGA AAT TGA TTA GT-3′)/C1N_2191 (5’-CAG GTA AAA TTA AAA TAT AAA CTT CTC G-3′) [34]. These primers amplify a fragment of approximately 680 and 470 bp of the cytochrome oxidase subunit 1 (*COI*) gene, respectively. Cycling conditions were as follows: initial denaturation at 95 °C for 5 min, followed by 35 cycles of denaturation (94 °C, 30 s), annealing (54 °C, 30 s), extension (72 °C, 120 s) and a final extension at 72 °C for 7 min.

For samples passing QC, *Rickettsia* presence was assayed using *Rickettsia*-specific primers amplifying (a) a section of the bacterial *16S rRNA* gene: Ri170_F (5′-GGG CTT GCT CTA AAT TAG TTA GT-3′)/Ri1500_R (5’-ACG TTA GCT CAC CAC CTT CAG G-3′) designed by Küchler et al. [17], and (b) the citrate synthase gene (*gltA)*; RiGltA405_F (5′-GAT CAT CCT ATG GCA-3′)/RiGltA1193_R (5’-TCT TTC CAT TGC CCC-3′) designed by Pilgrim et al. [16]. These primers have been shown to amplify across currently known *Rickettsia* groups but not cross amplify other alphaproteobacteria. Cycling conditions were the same as described above for the *COI*. Nuclease-free water was used as a negative control to ensure there were no false positive amplifications, and genomic DNA of *Culicoides newsteadi*, obtained from Pilgrim et al. [16] was used as a positive control. For each species where a positive amplicon was obtained, amplicons were cleaned of primer and unincorporated nucleotides, and Sanger sequenced from a subset of individuals. The obtained sequence was then used (a) to confirm that the amplicon was a *Rickettsia* gene product, and (b) to allow estimation of the relatedness of the strains found. These verified positive samples were also selected as positive control in subsequent screenings.

### Focussed Study of the UK Coenagrionid Species

Five additional UK coenagrionid species were collected from Cheshire, Hampshire and Norfolk. These samples were prepared and screened as described above to obtain *Rickettsia* sequences. Additionally, host mitochondrial barcodes were sequenced to confirm species identity, alongside additional markers to distinguish between the sister species *Coenagrion puella* and *C. pulchellum.* For distinction between *C. puella/pulchellum*, fragments of the Myosin light chain (MLC), Arginine methyltransferase (PRMT) and Phosphoglucose isomerase (PGI) nuclear genes were amplified and sequenced, following Ferreira et al. [32].

To allow a more in-depth study of *Rickettsia* diversity in the UK coenagrionid group, *Rickettsia* infections detected were further characterised by sequencing three additional loci; ATP-synthase (*atpA*), 17 kDa antigenic protein (*ompA*) and *COI* loci, to create a five loci allelic profile, allowing multi-locus sequence typing (MLST). The PCR conditions and primers used to amplify these genes were based on Pilgrim et al. [16].

Evidence for heritable symbiosis was investigated in *C. puella* by using fluorescence in situ hybridization (FISH) to ascertain the presence/absence of *Rickettsia* in ovarian tissues. Methods were adapted from Sakurai et al. [35]. Briefly, internal organs of three female *C. puella* (target species, *Rickettsia* positive) and three female *Ischnura elegans* (non-*Rickettsia*-infected species) were dissected and fixed in Carnoy’s solution (chloroform:ethanol:acetic acid, 6:3:1) overnight. Tissues were then cleared with 6% H_2_O_2_ in ethanol for 12 h or until the tissue were translucent (whichever was longer). Ovary material was then selected, and hybridisation conducted through incubating the tissues overnight in a hybridisation buffer (20 mM Tris-HCl pH 8.0, 0.9 M NaCl, 0.01% sodium dodecyl sulphate 30% formamide) with 10 pmol/ml of rickettsial rRNA-specific probe, 5'-CCA TCA TCC CCT ACT ACA-[ATTO 633]-3' [19]. After incubation, tissues were washed in buffer (0.3 M NaCl, 0.03 M sodium citrate, 0.01% sodium dodecyl sulphate), mounted onto a slide using VECTASHIELD® Antifade with DAPI as a mounting medium, and visualised under a confocal microscope (880 BioAFM).

### Diversity of *Rickettsia* Infections

The phylogenetic relatedness of *Rickettsia* strains found in odonates based on *16S rRNA* and *gltA* genes was estimated using MEGA X [36]. We selected several published sequences of *Rickettsia* from NCBI GenBank, including representatives varying in range from close to far distance relations to the strains in this study, based on BLAST homology. The far relative group consisted of several vertebrate pathogenic *Rickettsia* and other insect endosymbionts which are known belonging to other clades. *Occidentia massiliensis* was chosen as the outgroup for this *Rickettsia* topology. Sequences were manually checked and aligned using MUSCLE algorithm with default settings [37]. The relationships between these strains were estimated through the maximum likelihood approach using MEGA X, under the K2+I and T92+G+I model for *16S rRNA* and *gltA* gene, respectively. Support for individual nodes was tested with 1000 bootstrap replicates.

## Results

The initial broad screen of odonate material detected *Rickettsia* amplicons in 8 of the 76 species screened (Table 1), which represented nearly 50% of the families included in the screening. Positive material was derived from the UK, South America, mainland Europe and the Azores, indicating a broad geographic basis to the symbiosis. Four further *Rickettsia* symbioses were detected in the five additional UK species of Coenagrionidae tested in the focused screening (Table 1), resulting in a total of 6 of 8 UK coenagrionids testing positive.

In those cases where infection was detected in a species, the fraction of individuals testing positive for *Rickettsia* varied from 9 to 100% (Table 2). In two of the species with more than 1 sample, *C. puella* and *Enallagma cyathigerum*, 100% of the screened individuals were infected (Table 2). In cases where the individual sex was known (*i.e.*, template derived from adults), there was no evidence of *Rickettsia* infection being biased to one host sex (Table 2).

**Table 2:** Summary of *Rickettsia*-positive species, partitioned by host sex, identified across the broad and focused screens. Those listed as “unknown” correspond to non-sexed nymphs (n) and adults (*a*). Inside the brackets is the number of screened individuals, and outside is the number of infected individuals. Where multiple locations specified, the origin of the positive sample is marked with a superscript number indicating that the number of infected was found there. Asterisks indicate those UK coenagrionid species where the *Rickettsia* strains were successfully sequenced for all five MLST *loci*

No.	Species	Location	Male	Female	Unknown	% infected
UK						
**1**	Coenagrion puella*	Cheshire, UK	8 (8)	4 (4)	16 (16 n)	100
**2**	C. mercuriale*	Hampshire, UK	12 (20)	7 (10)	-	95
**3**	C. pulchellum*	Norfolk, UK	-	-	15 (20 a)	75
**4**	Enallagma cyathigerum*	Cheshire, UK	6(6)	1 (1)	-	100
**5**	*Erythromma najas*	Cheshire, UK	1 (5)	-	-	25
**6**	*Libellula depressa*	Cheshire, UK	-	-	1 (1 n)	100
**7**	Pyrrhosoma nymphula*	Cheshire, UK	2 (4)	2 (3)	-	57
South America						
**8**	*Drepanoneura muzoni*	Tiputini, Ecuador	1 (1)	0 (1)	-	50
**9**	*Neoneura sylvatica*	Minas Gerais, Brazil	1 (2)	-	-	50
**10**	*Polythore lamerceda*	Peru	0 (1)	1 (2)	-	33
**11**	*P. picta*	Pozuzo, Peru	1 (6)	0 (1)	-	14
Mainland Europe and the Azores						
**12**	*Sympetrum fonscolombii*	Azores, Portugal^2^ Villasimius, Italy	0 (6)	1 (4)	1 (12 n)	9

**Fig. 1:**
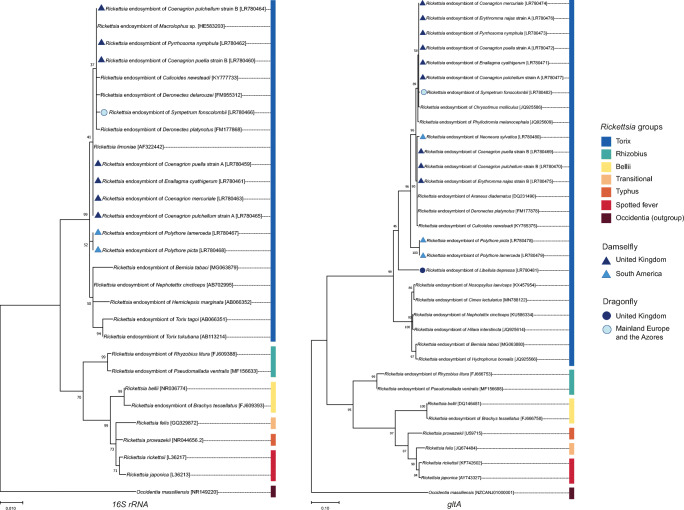
Phylogenetic analysis of torix *Rickettsia* based on *16S rRNA* and *gltA* gene sequences from screened odonate species, marked with coloured shapes, alongside reference DNA sequences of other *Rickettsia* groups obtained from GenBank (accession numbers in brackets). The tree was constructed in MEGA X by maximum likelihood, with K2+I and T92+G+I model for *16S* and *gltA*, respectively. Numbers above branches indicate bootstrap values from 1000 resampling events. Labels indicate the host species from which the symbiont amplicon was obtained

The *Rickettsia* strains from all 12 infected odonate species successfully produced *gltA* amplicons and *16S* amplicons could be observed from 9 of 12 infected species. All the sequenced amplicons were used in phylogenetic analysis, except the *Rickettsia* strain from *Drepanoneura muzoni*, which produced a low quality of DNA sequence for both genes (Fig. 1). The *Rickettsia* infections detected all belong to the torix subclade of *Rickettsia*. The infections were diverse, with multiple strains found in odonates, all of them closely allied to *Rickettsia* strains found in other invertebrate taxa (Fig. 1).

MLST of the *Rickettsia* infecting UK coenagerionid species revealed the presence of four closely related *Rickettsia* strains falling into two clusters, as established in the MLST profiles (Table 3). The data also revealed that the sister species *C. puella* and *C. pulchellum*, which share a mtDNA *COI* haplotype but are distinct at nuclear loci (data not shown), share two *Rickettsia* strains, A and B, (Table 3). In these two species, there was a mix of double (strain A and B) and single (only strain A) *Rickettsia*-infected damselflies (coinfection was observed in five of ten *C. puella*, and two of three *C. pulchellum*). There were no individuals of either species infected with single *Rickettsia* strain B. Focussed analysis of 10 *C. puella* and 3 *C. pulchellum* individuals revealed an individual was either repeatedly monomorphic, or repeatedly polymorphic, across five loci (five individuals of each type; see Supplementary Table). The polymorphisms observed were largely at synonymous sites, indicating retained functionality of the gene product.

**Table 3:** MLST allelic profiles of the *Rickettsia* found infecting five coenagrionid species from the UK. For any MLST gene locus, sequences with the same number are identical. A strain is defined as identity across all MLST *loci*

Species	MLST allelic profiles					
	*16S rRNA*	*gltA*	*ompA*	*atpA*	*coxA*	Strain
*Coenagrion puella* strain A	1	1	1	1	1	A
*C. puella* strain B	2	2	2	2	2	B
*C. pulchellum* strain A	1	1	1	1	1	A
*C. pulchellum* strain B	2	2	2	2	2	B
*C. mercuriale*	1	1	1	1	1	A
*Pyrrhosoma nymphula*	2	3	2	2	2	C
*Enallagma cyathigerum*	1	1	1	3	1	D

**Fig. 2:**
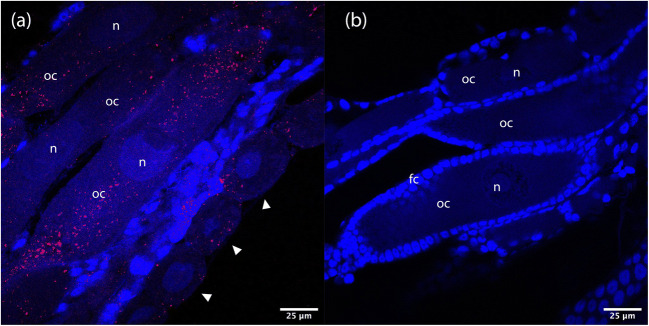
Fluorescence in situ hybridization (FISH) images showing the localisation of torix *Rickettsia* in **a** *C. puella* (*Rickettsia* positive) and **b** *I. elegans* (*Rickettsia* negative) oocytes. Red colour (ATTO633 label) represents *Rickettsia* signal and blue areas (DAPI) damselfly nuclei. Infection is observed throughout the ovary tissue of *C. puella*, mostly in oocytes (oc) and early differentiated oocytes (white arrowhead), but no signal of the symbiont was observed in the ovary of the *Rickettsia-*negative species, *I. elegans*; fc, follicular epithelial cells; n, nucleus of oocyte

The tissue-mounted fluorescence in situ hybridization revealed a cellular tropism of torix *Rickettsia* in *C. puella.* The signal of *Rickettsia* (ATTO-633 fluorophore) was detected throughout the ovary tissues of *C. puella*, mostly in the nuclei and cytoplasmic area of both mature and early developing oocytes, while the signal was absent in the non-infected species, *I. elegans* (Fig. 2).

## Discussion

There are numerous heritable microbe taxa that circulate in insects which play important roles as partners and antagonists. While the majority of studies have focused on the ‘global pandemic’ of *Wolbachia* and its consequences for host biology, ecology and evolution [38]; other heritable symbionts remain less well studied, particularly in freshwater insects. Here, we examined odonates for just one such symbiont—torix group *Rickettsia*.

Within the global screen, we detected *Rickettsia* in 8 of 76 odonate species (10.5%) and for the focussed UK screen, in 6 of 8 (75%) species from the coenagrionid family. The *Rickettsia* infections discovered all fall into the torix group, a basal group of *Rickettsia* with high levels of diversity, previously highlighted as common in other aquatic invertebrates [14, 16, 17]. The fraction of infected species in our screen is likely to be an underestimate, as there are two systematic biases likely to produce false negative results. First, symbiont infections usually vary in prevalence within species, and can infect a minority of individuals. The limited number of individuals tested for some of the species screened could therefore miss some species with low or intermediate levels of infection. Second, the material available for testing was commonly derived from legs. Symbiont infection that is strongly localised within a host individual (and not present in hemocytes) will appear as negative when leg material is screened. Furthermore, although our data record more infections in species of the UK coenagrionids than elsewhere, this could also be a product of a greater sampling intensity. What is clear, however, is that whilst odonates are hosts to *Rickettsia*, and they carry torix group strains like other freshwater invertebrates, they do not appear to be a particular hotspot for *Rickettsia*, when compared with other freshwater insects [16].

The study of torix *Rickettsia*/insect symbioses is a relatively young field of research, with this diverse group only first described in 2002 [15]. Thus, despite now being known to be widespread, data on the biology of these symbioses is absent or extremely limited. For instance, within host titres are unknown, meaning that we do not know how many cells have to be present for us to be able to detect an infection. However, *Rickettsia* distribution in insect tissues are commonly diffuse, including haemocytes, Malpighian tubules, gut lining, and in oocytes, where they seem to invade through the follicular epithelium and, unusually, they have also been found in sperm [39].

The symbioses in our study were found in representative species from the two odonate suborders: Zygoptera (damselflies) and Anisoptera (dragonflies). These species belong to four different families from both Europe and South America (Tables 1 and 2). Sequence analysis revealed a wide diversity in *Rickettsia* infections, suggesting the *Rickettsia*-odonate symbiosis has multiple origins. The odonate *Rickettsia* grouped together with strains found in other host species e.g., *Deronectes* water beetle, *Araneus* orb-weaving spider, *Culicoides* biting midge and *Cimex* common bedbug (Fig. 1). There also appeared to be a hotspot in the UK coenagrionids, in which four MLST strains from two clusters were observed, with two of these strains present in several species. The MLST study of *Rickettsia* is a recent initiative, introduced by Pilgrim et al. in 2017 [16]. Therefore, more fine-scale comparisons between the *Rickettsia* strains in our study with those found in other insect orders are limited in scope, due to lack of multilocus data from other taxa. However, this geographically confined clade may reflect symbiont movement between co-occurring odonate species or derivation from a common local source [40].

The presence of double peaks in sequences of *Rickettsia* marker genes in *C. puella* and *C. pulchellum* provide evidence of coinfection, where a single individual carries two strains of *Rickettsia*. Individuals either show one sequence of strain A at all markers, or two sequences mixing of strain A and B at all markers (with two strains identified). Variable loci can either be the product of two infecting symbiont strains, or a single symbiont alongside a symbiont genome insertion into the insect chromosome [41]. That the amplicons represent two symbionts, rather than a symbiont and a nuclear insertion of symbiont genetic material, is implied by the nature of the variants. The majority of variable sites observed are synonymous differences (e.g., in *GltA* gene has 16 SNP in 715 bps, of which 14 are synonymous and 2 non-synonymous) that indicate retained functionality of the gene. Retained functionality is expected for a symbiont copy (where function is required) rather than a nuclear insert (which is expected to pseudogenize). Coinfections are well known for *Wolbachia* [42] but are less commonly recorded for other symbionts; however, they are clear in this system.

Within the UK group, we observed a pair of *Rickettsia* strains shared by the sister species pair *C. puella* and *C. pulchellum*. This species pair is robustly supported in analysis of nuclear markers [32, 43], but shares a mtDNA barcode [40]. Shared mtDNA barcodes for otherwise distinct species pairs commonly reflects introgression of the mtDNA across the species boundary [44]. This process is known to be driven by *Wolbachia* in other cases [45, 46]. Whilst hybridization is considered very uncommon between these species [47], mitochondrial introgression requires only a single hybridization event, and it is likely that the shared mtDNA and symbiont in this case reflect a history of symbiont movement across the species barrier, along with accompanying mtDNA. This process produces distinct species, divergent at nuclear markers, that have no mtDNA ‘barcoding gap’, as observed in the case of *C. puella* and *C. pulchellum*. An implication of our results is that screening for *Wolbachia* alone is not sufficient to rule out symbiont-mediated introgression of mtDNA.

Torix *Rickettsia* are considered likely to show maternal inheritance, and in some cases also show paternal transmission [39]. In our system, *Rickettsia* were visible in *C. puella* ovarioles under FISH microscopy, making maternal inheritance very likely. Additionally, infection was detected in both larvae and adults, which implies vertical transmission (Table 2). Thus, our data supports the idea *Rickettsia* is a heritable symbiont in odonates, as inferred for other taxa [16, 39, 48, 49].

The significance of the symbiosis is uncertain. Vertical transmission through eggs ties *Rickettsia* transmission to odonate survival and reproduction, and thus selects for symbiont contribution to host function. Heritable symbionts are commonly important contributors to organismal function but the impact of torix *Rickettsia* on their host is unknown in all but one system. In the parasitoid wasp, *Pnigalio soemius* [3], torix *Rickettsia* are associated with the induction of parthenogenesis. However, sex-ratio distortion mediated by *Rickettsia* is unlikely in the case of odonates, as there were no obvious male/female host biases in species where large numbers of individuals were collected. Indeed, the symbionts were absent in the only odonate species known to have thelytokous parthenogenesis (*I. hastata* from the Azores islands) [50]. These data, by exclusion, indicate that symbionts are likely retained in odonate hosts by some other means, which should be explored further.

## Electronic supplementary material

ESM 1 (DOCX 37 kb)
