# Supplementary material for: Incidence and Diversity of Torix Rickettsia–Odonata Symbioses
Source: Microb Ecol. 2020 Aug 7;81(1):203–12. doi: 10.1007/s00248-020-01568-9 (PMC7794209; doi:10.1007/s00248-020-01568-9)
Supplement: Supplementary file 1 — (DOCX 37 kb) [file 248_2020_1568_MOESM1_ESM.docx]

**Supplementary table**

SNP sites across MLST genes from 10 *Coenagrion puella* and 3 *C. pulchellum* individuals, with non-synonymous substitution sites marked in bold. Non-synonymous/synonymous substitutions of each gene are in the bracket.

| *atpA* | | | | | | | | | | | | | | | | | |
| --- | --- | --- | --- | --- | --- | --- | --- | --- | --- | --- | --- | --- | --- | --- | --- | --- | --- |
| 660 bps (12/38) | | | | | | | | | | | | | | | | | |
| ID | Variable sites | | | | | | | | | | | | | | | | |
|  | 4 | 16 | 25 | 28 | 34 | 37 | **43** | **59** | **65** | **68** | 70 | **75** | **79** | 91 | 94 | 97 | 106 |
| *C. puella* | | | | | | | | | | | | | | | | | |
| 1 | T | T | C | G | A | A | **G** | **T** | **C** | **A** | T | **A** | **C** | G | T | C | T |
| 2 | C/T | C/T | C/T | A/G | A/C | A/G | **C/G** | **G/T** | **A/C** | **A/G** | C/T | **A/C** | **C/G** | A/G | C/T | C/T | C/T |
| 3 | T | T | C | G | A | A | **G** | **T** | **C** | **A** | T | **A** | **C** | G | T | C | T |
| 4 | C/T | C/T | C/T | A/G | A/C | A/G | **C/G** | **G/T** | **A/C** | **A/G** | C/T | **A/C** | **C/G** | A/G | C/T | C/T | C/T |
| 5 | C/T | C/T | C/T | A/G | A/C | A/G | **C/G** | **G/T** | **A/C** | **A/G** | C/T | **A/C** | **C/G** | A/G | C/T | C/T | C/T |
| 6 | T | T | C | G | A | A | **G** | **T** | **C** | **A** | T | **A** | **C** | G | T | C | T |
| 7 | T | T | C | G | A | A | **G** | **T** | **C** | **A** | T | **A** | **C** | G | T | C | T |
| 8 | T | T | C | G | A | A | **G** | **T** | **C** | **A** | T | **A** | **C** | G | T | C | T |
| 9 | C/T | C/T | C/T | A/G | A/C | A/G | **C/G** | **G/T** | **A/C** | **A/G** | C/T | **A/C** | **C/G** | A/G | C/T | C/T | C/T |
| 10 | C/T | C/T | C/T | A/G | A/C | A/G | **C/G** | **G/T** | **A/C** | **A/G** | C/T | **A/C** | **C/G** | A/G | C/T | C/T | C/T |
| *C. pulchellum* | | | | | | | | | | | | | | | | | |
| 1 | T | T | C | G | A | A | **G** | **T** | **C** | **A** | T | **A** | **C** | G | T | C | T |
| 2 | C/T | C/T | C/T | A/G | A/C | A/G | **C/G** | **G/T** | **A/C** | **A/G** | C/T | **A/C** | **C/G** | A/G | C/T | C/T | C/T |
| 3 | C/T | C/T | C/T | A/G | A/C | A/G | **C/G** | **G/T** | **A/C** | **A/G** | C/T | **A/C** | **C/G** | A/G | C/T | C/T | C/T |
| ID | 109 | 112 | 128 | 130 | 137 | 175 | 184 | 223 | **224** | **225** | **226** | 250 | **276** | **277** | **284** | 289 | 307 |
| *C. puella* | | | | | | | | | | | | | | | | | |
| 1 | T | C | A | A | C | G | C | T | **G** | **G** | **T** | T | **G** | **G** | **G** | A | G |
| 2 | C/T | C/T | A/C | A/G | A/C | G/T | C/T | C/T | **A/G** | **A/G** | **C/T** | C/T | **A/G** | **A/G** | **A/G** | A/T | A/G |
| 3 | T | C | A | A | C | G | C | T | **G** | **G** | **T** | T | **G** | **G** | **G** | A | G |
| 4 | C/T | C/T | A/C | A/G | A/C | G/T | C/T | C/T | **A/G** | **A/G** | **C/T** | C/T | **A/G** | **A/G** | **A/G** | A/T | A/G |
| 5 | C/T | C/T | A/C | A/G | A/C | G/T | C/T | C/T | **A/G** | **A/G** | **C/T** | C/T | **A/G** | **A/G** | **A/G** | A/T | A/G |
| 6 | T | C | A | A | C | G | C | T | **G** | **G** | **T** | T | **G** | **G** | **G** | A | G |
| 7 | T | C | A | A | C | G | C | T | **G** | **G** | **T** | T | **G** | **G** | **G** | A | G |
| 8 | T | C | A | A | C | G | C | T | **G** | **G** | **T** | T | **G** | **G** | **G** | A | G |
| 9 | C/T | C/T | A/C | A/G | A/C | G/T | C/T | C/T | **A/G** | **A/G** | **C/T** | C/T | **A/G** | **A/G** | **A/G** | A/T | A/G |
| 10 | C/T | C/T | A/C | A/G | A/C | G/T | C/T | C/T | **A/G** | **A/G** | **C/T** | C/T | **A/G** | **A/G** | **A/G** | A/T | A/G |
| *C. pulchellum* | | | | | | | | | | | | | | | | | |
| 1 | T | C | A | A | C | G | C | T | **G** | **G** | **T** | T | **G** | **G** | **G** | A | G |
| 2 | C/T | C/T | A/C | A/G | A/C | G/T | C/T | C/T | **A/G** | **A/G** | **C/T** | C/T | **A/G** | **A/G** | **A/G** | A/T | A/G |
| 3 | C/T | C/T | A/C | A/G | A/C | G/T | C/T | C/T | **A/G** | **A/G** | **C/T** | C/T | **A/G** | **A/G** | **A/G** | A/T | A/G |
| ID | 319 | 322 | 343 | 346 | 349 | 373 | 397 | 406 | 409 | 418 | 430 | 440 | 445 | 517 | 601 | 652 |  |
| *C. puella* | | | | | | | | | | | | | | | | | |
| 1 | G | T | T | A | C | G | C | T | T | T | C | T | T | G | G | T |  |
| 2 | A/G | A/T | A/T | A/C | C/T | A/G | C/T | A/T | C/T | C/T | C/T | C/T | G/T | A/G | G/T | C/T |  |
| 3 | G | T | T | A | C | G | C | T | T | T | C | T | T | G | G | T |  |
| 4 | A/G | A/T | A/T | A/C | C/T | A/G | C/T | A/T | C/T | C/T | C/T | C/T | G/T | A/G | G/T | C/T |  |
| 5 | A/G | A/T | A/T | A/C | C/T | A/G | C/T | A/T | C/T | C/T | C/T | C/T | G/T | A/G | G/T | C/T |  |
| 6 | G | T | T | A | C | G | C | T | T | T | T | T | T | G | G | T |  |
| 7 | G | T | T | A | C | G | C | T | T | T | T | T | T | G | G | T |  |
| 8 | G | T | T | A | C | G | C | T | T | T | T | T | T | G | G | T |  |
| 9 | A/G | A/T | A/T | A/C | C/T | A/G | C/T | A/T | C/T | C/T | C/T | C/T | G/T | A/G | G/T | C/T |  |
| 10 | A/G | A/T | A/T | A/C | C/T | A/G | C/T | A/T | C/T | C/T | C/T | C/T | G/T | A/G | G/T | C/T |  |
| *C. pulchellum* | | | | | | | | | | | | | | | | | |
| 1 | G | T | T | A | C | G | C | T | T | T | T | T | T | G | G | T |  |
| 2 | A/G | A/T | A/T | A/C | C/T | A/G | C/T | A/T | C/T | C/T | C/T | C/T | G/T | A/G | G/T | C/T |  |
| 3 | A/G | A/T | A/T | A/C | C/T | A/G | C/T | A/T | C/T | C/T | C/T | C/T | G/T | A/G | G/T | C/T |  |
|  | *16S* | *gltA* | | | | | | | | | | | | | | | |
|  | 1029 bp | 715 bp (2/14) | | | | | | | | | | | | | | | |
| ID |  | Variable sites | | | | | | | | | | | | | | | |
|  | 15 | 27 | 33 | **40** | **74** | 123 | 129 | 130 | 192 | 216 | 228 | 234 | 246 | 296 | 390 | 444 | 453 |
| *C. puella* | | | | | | | | | | | | | | | | | |
| 1 | A | A | A | **T** | **C** | T | T | C | G | A | C | T | C | T | G | T | T |
| 2 | A/G | A/G | A/G | **G/T** | **C/T** | C/T | C/T | C/T | A/G | A/G | C/T | C/T | C/T | A/T | A/G | C/T | C/T |
| 3 | A | A | A | **T** | **C** | T | T | C | G | A | C | T | C | T | G | T | T |
| 4 | A/G | A/G | A/G | **G/T** | **C/T** | C/T | C/T | C/T | A/G | A/G | C/T | C/T | C/T | A/T | A/G | C/T | C/T |
| 5 | A/G | A/G | A/G | **G/T** | **C/T** | C/T | C/T | C/T | A/G | A/G | C/T | C/T | C/T | A/T | A/G | C/T | C/T |
| 6 | A | A | A | **T** | **C** | T | T | C | G | A | C | T | C | T | G | T | T |
| 7 | A | A | A | **T** | **C** | T | T | C | G | A | C | T | C | T | G | T | T |
| 8 | A | A | A | **T** | **C** | T | T | C | G | A | C | T | C | T | G | T | T |
| 9 | A/G | A/G | A/G | **G/T** | **C/T** | C/T | C/T | C/T | A/G | A/G | C/T | C/T | C/T | A/T | A/G | C/T | C/T |
| 10 | A/G | A/G | A/G | **G/T** | **C/T** | C/T | C/T | C/T | A/G | A/G | C/T | C/T | C/T | A/T | A/G | C/T | C/T |
| *C. pulchellum* | | | | | | | | | | | | | | | | | |
| 1 | A | A | A | **T** | **C** | T | T | C | G | A | C | T | C | T | G | T | T |
| 2 | A/G | A/G | A/G | **G/T** | **C/T** | C/T | C/T | C/T | A/G | A/G | C/T | C/T | C/T | A/T | A/G | C/T | C/T |
| 3 | A/G | A/G | A/G | **G/T** | **C/T** | C/T | C/T | C/T | A/G | A/G | C/T | C/T | C/T | A/T | A/G | C/T | C/T |
|  | *CoxA* | | | | | *OmpA* | |  |  |  |  |  |  |  |  |  |  |
|  | 624 bp (0/5) | | | | | 242 bp (2/2) | |  |  |  |  |  |  |  |  |  |  |
| ID | Variable sites | | | | | | |  |  |  |  |  |  |  |  |  |  |
|  | 104 | 143 | 246 | 264 | 311 | **64** | **235** |  |  |  |  |  |  |  |  |  |  |
| *C. puella* | | | | | | | |  |  |  |  |  |  |  |  |  |  |
| 1 | A | A | C | C | G | **G** | **A** |  |  |  |  |  |  |  |  |  |  |
| 2 | A/G | A/G | C/T | C/T | A/G | **A/G** | **A/G** |  |  |  |  |  |  |  |  |  |  |
| 3 | A | A | C | C | G | **G** | **A** |  |  |  |  |  |  |  |  |  |  |
| 4 | A/G | A/G | C/T | C/T | A/G | **A/G** | **A/G** |  |  |  |  |  |  |  |  |  |  |
| 5 | A/G | A/G | C/T | C/T | A/G | **A/G** | **A/G** |  |  |  |  |  |  |  |  |  |  |
| 6 | A | A | C | C | G | **G** | **A** |  |  |  |  |  |  |  |  |  |  |
| 7 | A | A | C | C | G | **G** | **A** |  |  |  |  |  |  |  |  |  |  |
| 8 | A | A | C | C | G | **G** | **A** |  |  |  |  |  |  |  |  |  |  |
| 9 | A/G | A/G | C/T | C/T | A/G | **A/G** | **A/G** |  |  |  |  |  |  |  |  |  |  |
| 10 | A/G | A/G | C/T | C/T | A/G | **A/G** | **A/G** |  |  |  |  |  |  |  |  |  |  |
| *C. pulchellum* | | | | | | | |  |  |  |  |  |  |  |  |  |  |
| 1 | A | A | C | C | G | **G** | **A** |  |  |  |  |  |  |  |  |  |  |
| 2 | A/G | A/G | C/T | C/T | A/G | **A/G** | **A/G** |  |  |  |  |  |  |  |  |  |  |
| 3 | A/G | A/G | C/T | C/T | A/G | **A/G** | **A/G** |  |  |  |  |  |  |  |  |  |  |
